# TubZ filament assembly dynamics requires the flexible C-terminal tail

**DOI:** 10.1038/srep43342

**Published:** 2017-02-23

**Authors:** Maria E. Fuentes-Pérez, Rafael Núñez-Ramírez, Alejandro Martín-González, David Juan-Rodríguez, Oscar Llorca, Fernando Moreno-Herrero, Maria A. Oliva

**Affiliations:** 1 CSIC – Centro Nacional de Biotecnología, Department of Macromolecular Structures, Cantoblanco, Madrid, 28049, Spain; 2 Department of Medicine, Section of Virology, Imperial College, Du Cane Road, London, W120NN, UK; 3 CSIC – Centro de Investigaciones Biológicas, Department of Chemical and Physical Biology, Madrid, 28040, Spain

## Abstract

Cytomotive filaments are essential for the spatial organization in cells, showing a dynamic behavior based on nucleotide hydrolysis. TubZ is a tubulin-like protein that functions in extrachromosomal DNA movement within bacteria. TubZ filaments grow in a helical fashion following treadmilling or dynamic instability, although the underlying mechanism is unclear. We have unraveled the molecular basis for filament assembly and dynamics combining electron and atomic force microscopy and biochemical analyses. Our findings suggest that GTP caps retain the filament helical structure and hydrolysis triggers filament stiffening upon disassembly. We show that the TubZ C-terminal tail is an unstructured domain that fulfills multiple functions contributing to the filament helical arrangement, the polymer remodeling into tubulin-like rings and the full disassembly process. This C-terminal tail displays the binding site for partner proteins and we report how it modulates the interaction of the regulator protein TubY.

Tubulin-like GTPases play a central role in a wide range of physiological processes in cells, plasmids and viruses through their assembly into dynamic cytomotive filaments. In the presence of GTP, these proteins assemble in a head-to-tail fashion and the addition of new filament molecules involves the formation of the complete GTPase active site^1^. During filament assembly, GTP hydrolysis induces a conformational change that makes filaments prone to depolymerization. Further, there are structural differences between unassembled and assembled monomers unrelated to the nucleotide chemical state, known as structural plasticity, that are key in the modulation of the polymer dynamics^2^.

TubZ is a GTPase of the tubulin superfamily that functions as the motor component of the DNA positioning system by forming a spindle-like apparatus^3^. These segregation systems are the survival kits of *Bacillus* and *Clostridium* virulence plasmids that ensure their faithful inheritance by daughter cells during division. The plasmid partitioning requires a cis-acting centromere-like DNA sequence (*tubC*) and a centromere binding protein (TubR) that collectively form the partitioning complex. TubZ interacts with this complex and elicits plasmid trafficking along the cytoplasm^4^. Furthermore, there is another protein, TubY, that can bind to and modify the dynamics of TubZ filaments^5^. TubY can also bind to the operon promoter and activate transcription^6^. The understanding of plasmid segregation is key in order to design new strategies to prevent the spread of virulence genes in growing bacterial populations. Both TubZ and the regulator protein TubY may be ideal targets to block DNA movement within growing bacteria. However, in order to develop novel and specific drugs to target plasmid segregation we need a better understanding of their mechanism of action.

In microtubules, tubulin dimers associate into a tubular-like structure where the continuous addition of GTP-containing tubulin subunits forms caps that stabilize the structure^7^. Loss of these caps results in rapid depolymerization known as dynamic instability^8^. The consensus view agrees that during depolymerization a straight to curved structural change in microtubule protofilaments occurs^9^. However, in the presence of these caps microtubules can undergo treadmilling dynamics^10^. Like tubulin, TubZ forms polar assemblies that can follow dynamic instability^11^ and treadmilling^3^,^12^. However, TubZ polymerizes in a helical fashion, suggesting that the conformational changes associated with nucleotide hydrolysis and the structural plasticity may be different. Recent cryo-EM structures of 2- and 4-stranded TubZ filaments showed an opening in the longitudinal interface due to GTPase activity^13^. Further, these structures displayed contacts between the C-terminal tail and upper TubZ monomers within the protofilament, however a lack in continuous electron density made it impossible to describe the interaction mechanism^13^. Herein, we have analyzed TubZ filament structure with and without GTP caps when considering the full-length protein and C-tail truncations to gain insight into the molecular mechanism of filament dynamics. Our structural and biochemical data reveal that GTP caps retain the helical pattern and that GTP hydrolysis produces an unexpected filament stiffening upon disassembly. Further, the flexible C-tail participates as a multifunctional domain on filament assembly and disassembly.

## Results

### CbTubZ primarily assembles as a 4-stranded helical filament

According to cryo-EM reconstructions of *Bacillus thuringiensis* TubZ (BtTubZ) polymers, Montabana and Agard^13^ proposed that structural changes in TubZ filaments during nucleotide hydrolysis involve an increase from 2 (GTP- γ-S-bound) to 4 (GDP/GTP-bound) protofilaments. We have measured the nucleotide content of BtTubZ filaments assembled with GTP- γ-S and GTP and have found 20% and 80% of GDP-bound molecules, respectively (Methods). Therefore, we wanted to investigate whether the GDP content was necessary to induce such conformational changes.

**Figure 1:**
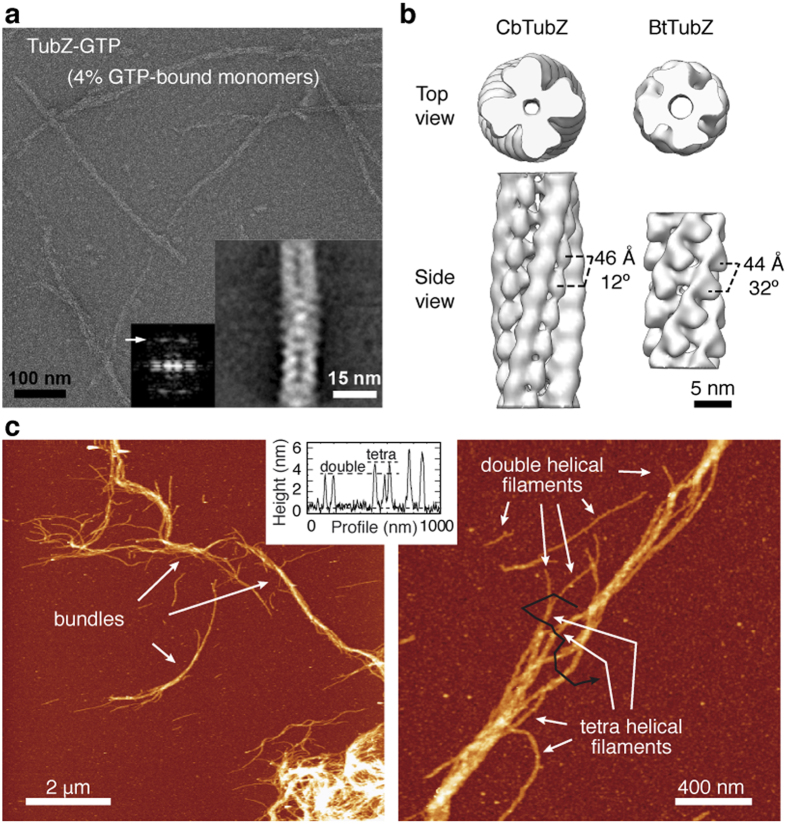
Characterization of TubZ filaments. (**a**) Negative stain EM showing wild type CbTubZ filaments. Inset corresponds to the averaged filament and its Fourier transform. Arrow indicates a 4.8-nm longitudinal spacing between molecules. (**b**) Top and side views of our CbTubZ low-resolution filament versus BtTubZ filament^13^ filtered at a similar low-resolution level. Both filaments are 4-stranded but show different longitudinal distances between monomers and azimuthal angles. (**c**) AFM images of CbTubZ_T100A_ filaments, showing 2 – and 4-stranded filaments and bundles. The black line in the second image corresponds to the height profile shown in the graph. Color scale from dark to bright corresponds to 0–11 nm.

To better understand the architecture of TubZ filaments and how its nucleotide-bound state affects its structure, we analyzed wild type *Clostridium botulinum* TubZ (CbTubZ) polymers and mutated versions at the nucleotide-binding site (CbTubZ_T100A_) and the catalytic loop (CbTubZ_E200A_). These proteins assembled in the presence of GTP and GTP- γ-S, but the resulting filaments showed different hydrolysis ratios^5^. Further, the nucleotide content and the size of the defined polymer’s caps^14^ differed considerably. For the wild type protein, 4% of GTP-bound molecules were observed while up to 20% and 80% GTP-bound molecules were observed for the CbTubZ_T100A_ and CbTubZ_E200A_ mutants, respectively^5^. We combined electron microscopy (EM) and atomic force microscopy (AFM) to get an average projection of the filaments and an accurate measurement of their heights that provided useful information on polymer 3D structure. Surprisingly, the filaments analyzed yielded similar averaged structures including those grown in the presence of GTP- γ-S (Fig. 1a and Fig. S1a). The filaments shared a 4-stranded helical arrangement with a rise of ~46 Å and an azimuthal angle of ~12° (Fig. 1b and Fig. S1b). These filaments had mean heights of 3.2 ± 0.4 nm (measured from a mean basal line), and mean widths of 27 ± 4 nm (measured as the full-width at half-maximum height) (N > 50, Fig. 1c). However, mean widths of 13.6 nm were deduced after the correction of the tip-convolution effect when considering the tip size of 10 nm (as provided by Nanosensor, Switzerland). This value is slightly lower than that obtained in previous cryo-EM reconstructions^13^, probably due to a dehydration effect on AFM samples when imaged in air^15^. By contrast, BtTubZ 4-stranded filaments have a rise of ~44 Å and an azimuthal angle of ~32°^13^, indicating that despite sharing a common core^5^,^16^, CbTubZ and BtTubZ display structural differences that underlie distinct helical patterns. In our images we also identified thinner filaments with mean heights of 2.7 ± 0.3 nm and mean widths of 16 ± 3 nm (5.6 nm after correction) that likely correspond to 2-stranded forms (Fig. 1c and Fig. S2a)^5^, however we were unable to obtain acceptable EM averages due to their low frequency.

It may be argued that *in vitro* filament assembly could differ from cell-based filament assembly. One explanation for this could be differences within the cellular environment such as macromolecular crowding. Moreover, higher viscosity and the excluded volume effect would increase the likelihood of intermolecular interactions^17^. Differences in the filament assembly of tubulin-like FtsZ polymers have been described in diluted versus crowded conditions^18^. To simulate the crowded cytoplasm of a bacterium we studied TubZ polymerization using Ficoll-70, a chemically inert sucrose-based polymer that occupies space in order to mimic molecular crowding but does not interact with the protein^19^. In these conditions, TubZ filaments retained their helical structure displaying widths corresponding to 2-stranded (7.7 ± 0.7 nm) and 4-stranded (13.7 ± 2.2 nm) polymers (N > 50, Fig. S2a,b), in agreement within the error of AFM measurements. However, filament dynamics decreased significantly in the presence of Ficoll (Fig. S2c).

Our findings strongly suggest that the functional CbTubZ polymer is a 4-stranded filament and that the 2-stranded filament may constitute an intermediate stage during polymerization that rapidly moves toward a 4-stranded form, regardless of nucleotide content. By contrast, BtTubZ is 2-stranded filament when assembled with GTP- γ-S^13^,^20^. Hence, GTP- γ-S might blocks BtTubZ filaments on the double helical state, which could be a non-functional filament during DNA trafficking.

### GTP caps are essential for maintenance of 4-stranded filament morphology

**Figure 2:**
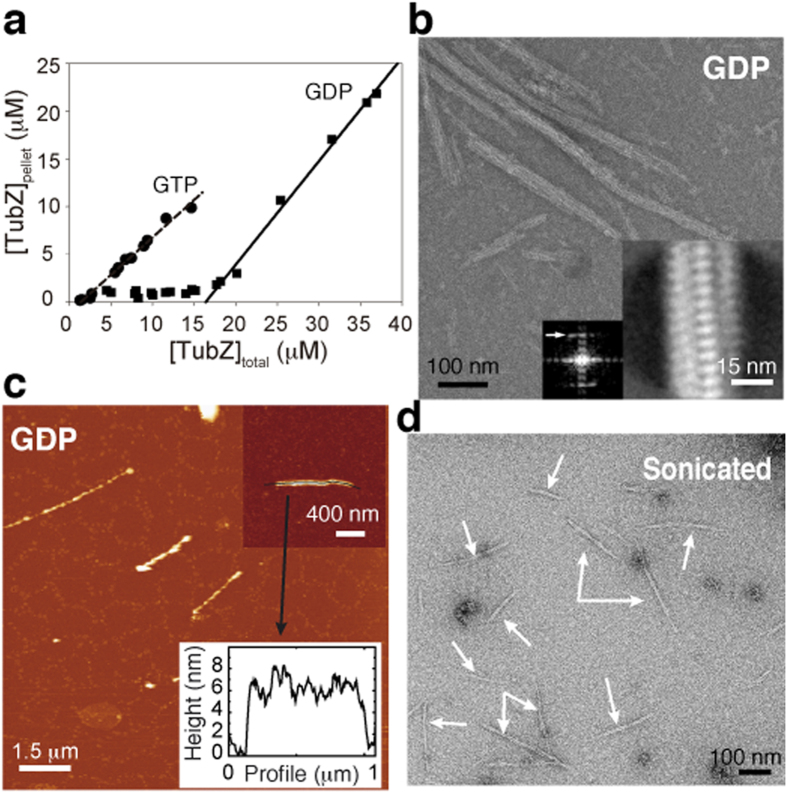
Effect of GTP hydrolysis on filament structure. (**a**) Critical concentration measurements of CbTubZ in buffer containing Ficoll (200 g/L) and GTP/Mg^2+^ or GDP/Mg^2+^ (**b**). Negative stain EM showing CbTubZ filaments polymerized in the presence of GDP/Mg^2+^. Inset shows averaged filaments and the corresponding Fourier transform. Arrow indicates 4.8-nm longitudinal spacing between molecules. (**c**) AFM images of CbTubZ filaments polymerized in the presence of GDP/Mg^2+^. The black line corresponds to the height profile shown in the graph. Color scale from dark to bright correspond to 0–11 nm. (**d**) Negative stain EM of CbTubZ filaments assembled with GTP/Mg^2+^ and sonicated for 10 min, showing the stiffening effect.

To unravel the molecular mechanism underlying filament dynamics, we analyzed the effect of GTP caps release. First, we forced the formation of pure GDP-bound filaments lacking GTP caps by supplying only GDP during the assembly reaction. Despite a lower affinity between monomers (higher critical concentration), we found that CbTubZ assembled cooperatively onto stable filaments (Fig. 2a). However, the polymer structure was different than the 4-stranded filament described above, showing both wider (20–40 nm) and thicker (5–8 nm) dimensions (Fig. 2b,c). Furthermore, the resulting filament was straight and appeared rigid, but retained the intermonomer spacing (measured from the averaged image Fourier transform) and some degree of helical trend (Fig. 2b).

Next, we analyzed the effect of removing 4% of GTP-bound molecules that stabilize CbTubZ filaments (GTP caps). To this end, we polymerized TubZ in a solution containing an excess of GTP and Mg^2+^ and examined the resulting filaments after disrupting them by mechanical force (sonication). We identified multiple straight and short filaments as breakdown products of GTP-formed TubZ filaments (Fig. 2d). Since CbTubZ polymer is mainly GDP-bound, it is reasonable to assign these short products as the GDP-bound state.

We hypothesize that in the presence of GTP caps, lattice contacts may control and maintain the 4-stranded helical arrangement, similar to what has been described for microtubules^21^. Unlike tubulin, however, there is no protofilament curving, but rather filament stiffening, thereby promoting its disassembly. In TubZ, protofilaments are helical and the straightening induced by GTP hydrolysis may generate the necessary forces to break longitudinal contacts. Here, fewer contacts would make a weaker interface favoring monomer detaching.

### Role of the C-tail during the assembly process

**Figure 3:**
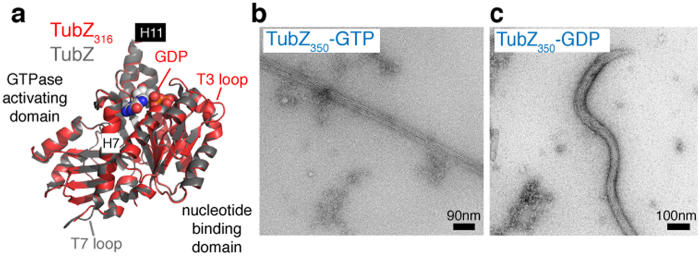
Effect of C-tail on TubZ assembly. (**a**) Comparison of full length and CbTubZ_316_ structures, highlighting the main domain arrangements and the most important secondary structure elements. TubZ_316_ displays GDP at the nucleotide-binding pocket, an ordered T3 loop and a disordered T7 loop (which is ordered in the full-length protein). In both structures the C-terminal helix H11 is in the same position, but neither contain an ordered C-tail. (**b**,**c**) Negative stain EM of CbTubZ_350_ stiff filament assembled in the presence of GTP/Mg^2+^ (**b**) and flexible filament polymerized with GDP/Mg^2+^ (**c**).

The C-tail of TubZ is flexible and ranges in length and amino acid composition from short (~20 residues) and acidic to long (more than 60 residues) and basic^16^,^22^. Since the C-tail makes contacts with upper subunits within the filament during polymerization^13^,^23^, we further investigated its functional implication. The C-tail of CbTubZ is long (50 residues) and acidic, so we generated three truncated mutants: CbTubZ_350_, lacking the last 8 residues that are not charged; CbTubZ_331_, lacking the same residues as for CbTubZ_350_ plus an acidic patch and CbTubZ_316_, lacking the entire flexible tail (Fig. S3a). All three constructs were expressed and purified as soluble proteins at similar levels as the wild type protein. Similarly, all showed equivalent circular dichroism spectra, suggesting that the truncations did not affect the core structure (Fig. S3b). Further, we solved the crystal structure of CbTubZ_316_ at 2.4 Å resolution (PDB 4XCQ, Table S1), which closely superposed with the apo full-length protein (PDB 3V3T) with an r.m.s.d of 0.37 Å (Fig. 3a). Moreover, the nucleotide-binding affinities of these mutants were similar to those of the wild type protein (5–6 × 10^6^ M^−1^, Fig. S3c), suggesting that the nucleotide-binding step is not limiting during the polymerization process.

Similar to other C-tail truncated TubZs, our CbTubZ mutants showed no assembly in diluted solutions^13^,^24^. However, based on the differing kinetics observed in crowded versus diluted conditions, we examined filament assembly with the mutated proteins in an *in vitro* crowded environment using Ficoll-70. While all three mutants polymerized cooperatively, the critical concentration increased progressively in parallel with the absence of longer sequences from the tail region (Fig. S3d), indicating reduced polymer elongation affinity as compared with the wild type protein. Although these mutants retained the ability to polymerize, none showed significant levels of GTPase activity (Fig. S3e), which may be related to a defect on the formation of the GTPase active site (i.e. the insertion of the catalytic loop in the GTP binding pocket of the lower subunit). Indeed, the assembled polymers no longer displayed a helical arrangement (Fig. 3b, Fig. S3f), instead they resembled the wild type GDP-bound filament (Fig. 2b). This suggests that the modulation of the C-tail tip interactions may contribute to the stiffening of the filament after hydrolysis. Further, the C-tail movement during GTP hydrolysis could provide a simple explanation for the clashes observed for TubZ-GDP C-tail, as described during its docking into the GTP-like (GMPCPP) cryoEM filament structure^23^.

In the head-to-tail assembly process the addition of a new monomer to the tip of the filament implies that the C-tail is probably free, because there are no upper subunits available to form additional contacts with the protein. Here, we show that by removing the very end of the C-tail (8 residues) the filament morphology changes dramatically. Therefore, during assembly those amino acids may make the initial contacts (with the most distant monomer in the row), after which the charged residues can participate in the stabilization of the tail (as previously described^23^) through interaction with more proximal molecules. Our results suggest that the C-tail domain is directly involved in modulating filament assembly as well as facilitating the formation of a functional helical filament. Differences in tail length may affect the degree of monomer rotation within the filament and hence its twist. In fact, BtTubZ (with more than 60 residues) and CbTubZ (with 50 residues) assemble into 4-stranded filaments with distinct helical pitches (above). By contrast, phages 201 ϕ2-1 and KZ TubZs (both with 20 residues) polymerize into 3-stranded filaments^23^ and have longer helical pitches (~51 nm)^25^.

We further wanted to understand how nucleotide hydrolysis induces depolymerization. In order to answer this question, we assembled truncated C-tail CbTubZs in the presence of GDP. The resulting filaments were curly rather than straight, denoting increased flexibility (Fig. 3c and Fig. S3g). This is probably due to a weakening of the longitudinal interactions between monomers as mention previously^13^. We hypothesize that this softening of the longitudinal interactions produced by GTP hydrolysis is the last step preceding subunit disassembly from the filament (see the proposed model below, Fig. 5).

### The C-tail modulates interactions with TubY and the formation of tubulin-like rings

**Figure 4:**
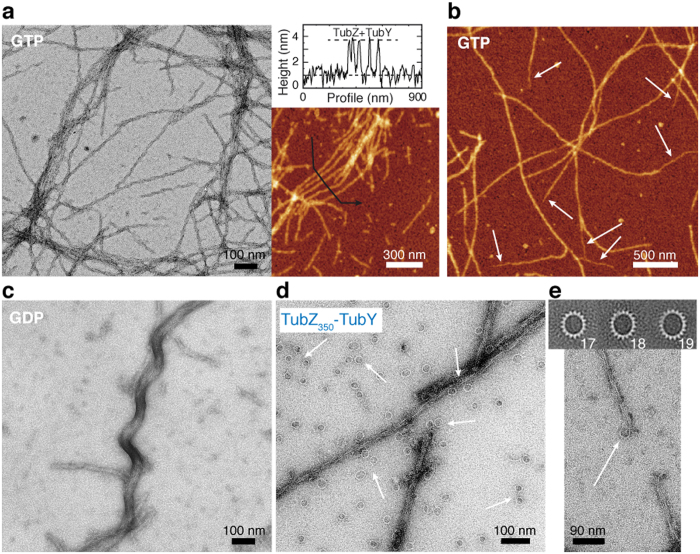
TubY - TubZ interaction. (**a**) Negative stain EM (left) and AFM (right) of CbTubZ-TubY filaments. The height profile shown in the graph was taken in the direction of the black arrow. (**b**) AFM images of CbTubZ_E200A_-TubY filaments. White arrows indicate thinner extensions at the ends of some filaments. Color scale from dark to bright is 0–11 nm in AFM images. (**c**–**e**) EM images of TubY binding to GDP-bound CbTubZ filaments (**c**), TubZ_350_ filaments and rings formed in the presence of TubY (**d**) and detail of ring formation upon disassembly from filaments tips (**e**, bottom). (**e**) Different types of averaged structures obtained after ring processing, including the number of monomers in each ring (top).

We previously demonstrated that TubY binding to TubZ affected filament dynamics, however we could not identify the underlying molecular mechanism^5^. Now we have analyzed the effect of filament nucleotide content on TubY binding. Sedimentation experiments revealed that TubY interacts similarly with wild type, CbTubZ_T100A_, and CbTubZ_E200A_ filaments (Fig. S4a). Furthermore, the resulting polymers were also similar according to EM and AFM images showing mean heights of 2.7 ± 0.4 nm (Fig. 4a). The height values measured were slightly lower than expected due to an increase in the basal plane caused by the presence of a greater quantity of protein absorbed on the surface (see Methods). Indeed, the amplitude of noise in the basal plane was 1.4 ± 0.4 nm as compared with 0.9 ± 0.2 nm when measured in the absence of TubY. Interestingly, we observed thinner extensions at the ends of some filaments (white arrows in Fig. 4b), with mean heights of 2.0 ± 0.2 nm, suggesting coating of TubZ filaments by TubY. Hence, the binding region is likely accessible along the whole filament, regardless of the nucleotide content. Surprisingly, TubY clearly modified the wild type GDP-bound polymer structure (Fig. 2b), which became more flexible and in turn resembled the GDP-bound filaments of C-tail truncated proteins (Figs 3c and 4c). This result supports a regulatory function of TubY in modulating the dynamics of TubZ filaments by modifying subunit interactions.

Considering our findings regarding the role of the C-tail in TubZ filament assembly and its interaction with TubR^16^, we examined the role of the C-tail in TubY binding. Light scattering experiments revealed interactions with all the truncated proteins used in this study (Fig. S4b). Interestingly, TubY binding induced the rearrangement of CbTubZ_350_ filaments into tubulin-like rings (Fig. 4d), suggesting that the C-tail domain may modulate this interaction. We previously described the formation of similar rings during TubY binding to TubZ filaments in the presence of the partition complex TubR*C*^5^. Thus, TubY and the segrosome may compete for the C-tail tip, and TubY may promote the filament re-arrangement into rings only when the TubR*C* complex blocks the TubZ C-terminal tip.

The formation of rings is a recurring subject in tubulins^26^,^27^,^28^. These rings are equivalent to coiled protofilaments with a certain degree of curvature. Their formation requires a significant remodeling of the subunit-subunit longitudinal and lateral contacts. In some images, we could observe ring-like structures “peeling off” at the ends of filaments (Fig. 4e and Fig. S4c), resembling typical images that can be observed for depolymerizing microtubules. This supports the view that contacts within the rings are related to the longitudinal contacts within the filament. Image processing of ~1200 independent rings revealed an inherent flexibility with ring sizes ranging from 17 to 19 monomers (rings diameter, 24–29 nm; Fig. 4e), as also described for tubulin and FtsZ rings^26^, suggesting a similar degree of inter-monomer bending. Taken together, our results show that despite marked differences (helical versus straight filaments), TubZ conserves inter-subunit interfaces and the bending ability of tubulin-like proteins.

## Discussion

**Figure 5:**
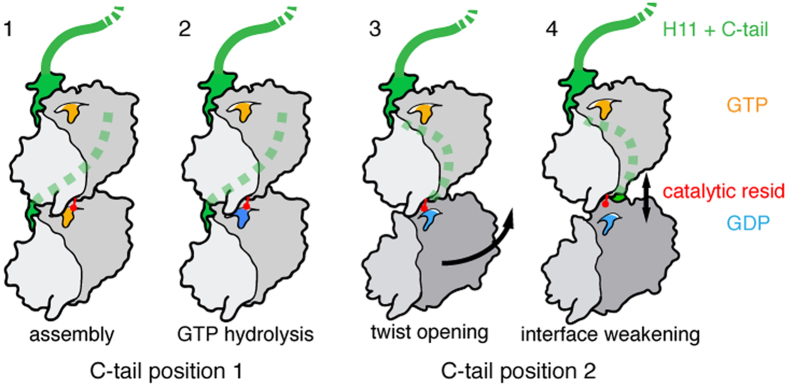
Model of TubZ filament dynamics. For simplicity, we show only two consecutive molecules in the filament: (1) GTP-bound state induces the assembly, whereas the C-tail (green and dashed line when behind a protein subunit) establishes specific contacts with upper subunits leading to the generation of a twist and allowing the formation of the canonical longitudinal interface; (2) the catalytic residue (red) is correctly positioned and reaches GTP (yellow), which is hydrolyzed into GDP (blue); (3) the enzymatic reaction induces changes in the bottom molecule, changing the C-tail interacting surface with the upper subunits and opening the twist between both molecules; (4) the GDP-bound state results in weakening of the longitudinal interface, likely due to loss of contacts. C-tail truncated proteins do not establish the specific contacts with upper subunits within the filament, blocking the formation of the canonical longitudinal interface. Accordingly, GTP assembly leads to state 3 filaments, while GDP-bound polymers are in state 4.

Unlike tubulin, TubZ molecules must rotate during polymerization to generate a helical filament. The correct formation of the longitudinal interface is paramount in order for hydrolysis to yield a dynamic filament. The relative rotation between the N- and C-terminal domains gives rise to a twist in the TubZ protofilament^20^. However, when 2, 3 or 4 protofilaments wrap around each other, the rotation must change to conserve the same longitudinal interface. Our findings suggest that the C-tail is a flexible domain that drives this change and enables the correct positioning of monomers; being responsible for the helical pitch and the number of protofilaments of the resulting assembly. Further, this domain is also involved in the filament stiffening upon disassembly. Based on our results and previous findings, we propose a model to explain TubZ filament dynamics (Fig. 5). TubZ in solution is prone to assemble upon GTP binding, as also described for tubulin^29^. The first interaction of the newly added TubZ molecule may result in a certain degree of rotation in the monomer^20^, but its positioning is inappropriate for GTP hydrolysis. Depending on the length of the C-tail, the addition of one or several molecules is necessary so the C-tail tip can make an initial contact, which is followed by the stabilization of the entire C-tail via the charged residues (Step 1). Once the molecule is correctly positioned and the catalytic loop reaches the terminal gamma phosphate, GTP hydrolysis can occurs (Step 2). GTPase activity triggers the opening of the twist in the longitudinal subunit-subunit interface^13^ and a rearrangement of the C-tail binding interactions with upper molecules happens (Step 3). At this point filament stiffening does not occur due to the stability of the filament lattice and the presence of GTP caps. However, once the GTP caps are lost a conformational change occurs that triggers depolymerization. Moreover, the longitudinal subunit-subunit interfaces become too weak to hold the molecules together (Step 4), leading to the release of those molecules during filament disassembly. In fact, the crystal structure of phages 201 ϕ2-1 and KZ TubZ (both in the presence of GDP) show a protofilament-like arrangement that may constitute two stages of the weakening process after GTP hydrolysis. In both structures, the C-tail is already in a post-hydrolysis state. However, KZ TubZ may represent an initial stage with a larger (999.6 Å^2^, versus 205 Å^2^ in 201ϕ2-1 TubZ) and more proximal interface area^25^ (i.e. the distance between the catalytic loop and GDP is 6.9 Å, versus 12.2 Å in 201ϕ2-1 TubZ). The proposed model can fit both treadmilling and dynamic instability. In the presence of a GTP cap, depolymerization occurs preferentially from the filament minus end, where the subunit-subunit interface may be weaker due to the inherent filament polarity. However, if the cap is lost, the entire filament becomes stiffer, thus triggering a complete disassembly.

The C-tail tip plays a dual role, on one hand, it modulates monomer binding with upper subunits within the filament and, on the other hand, it interacts with the segrosome (TubR*C*) and TubY, probably once the tail is stabilized through charged residues. Based on results showing that the segrosome only binds at the TubZ filament end^12^, we propose that although TubY binds to the whole filament, it only facilitates filament disassembly at the segrosome-attached end. Thereby, contributing to DNA movement along the bacteria cytoplasm.

Recent studies have proposed that tubulin and tubulin-like FtsZ rings display opposite curvatures when protofilaments bend to form a ring. This has been explained in terms of the different localization of the C-terminus within the ring^30^. Further studies will be required to elucidate the biological function of TubZ rings and their structural arrangement with regards to their bending orientation, the positioning of the C-tail and the presence of TubY during this filament rearrangement.

A key finding of our study is the importance of the TubZ C-tail on protein assembly and filament disassembly modulation. Since TubZ is the motor protein in plasmid encoded segregation systems, inhibiting its assembly or filament dynamics would in turn seriously affects DNA trafficking during bacterial cell division. Considering that high virulence *Bacillus* and *Clostridium* strains carry plasmids encoding this kind of partition system, perturbing the plasmid maintenance would lead to a completely lost of the bacteria toxicity. According to our results, the design of such new drugs would involve peptides mimicking different TubZ C-tails. Further studies are warranted that would answer the effectiveness of these kinds of compounds and their applicability in a population of growing bacteria.

## Methods

### Cloning, protein expression and purification

Wild type TubZ and the mutants TubZ_T100A_, and TubZ_E200A_, and TubY_226_ were cloned as previously described^5^. TubZ C-terminal truncated proteins were obtained by PCR mutagenesis using our wild type construct vector (a modified pET28 vector that includes an N-terminal His tag followed by a 3C protease site) as a template. All proteins were expressed and purified as previously described^5^ and stored at −80 °C. New constructs were analyzed by mass spectrometry.

### TubZ assembly

The time course of polymerization was monitored by right angle light scattering analysis of 0.5-mL samples at 350 nm (0.5 nm excitation and emission slits widths) at 30 °C, except for controls and AFM experiments (25 °C). Proteins were diluted in PKE assembly buffer (50 mM Pipes/KOH [pH 6.5], 100 mM potassium acetate, 1 mM EDTA) with or without 200 g/L Ficoll-70 (prepared in PKE buffer), and polymerization was initiated by the addition of nucleotide (1 mM GTP, 0.1 mM GTP- γ-S or 2 mM GDP) and 6 mM magnesium acetate. Polymer formation was quantified by sedimentation assays. Samples were prepared as described above and centrifuged for 30 min at 60,000 rpm in a TLA100 rotor. The supernatants were carefully withdrawn and the pellets were resuspended in the same volume of buffer and both samples were loaded in consecutive lanes of 12% polyacrylamide gels. Gels were stained with Coomassie Blue, scanned with a Bio-Rad CS-800 calibrated densitometer and analyzed with Quantity One software (Bio-Rad). Alternatively, protein concentration was measured with the Bio-Rad protein assay kit using spectrophotometrically calibrated TubZ standards for the calibration curve. The critical concentration was determined based on the relation between the amounts of protein in the pellet relative to the total amount of protein at different protein concentrations.

### Electron microscopy

Samples from polymerization experiments were adsorbed onto carbon-coated grids and negatively stained with 2% uranyl acetate. Images were taken at various magnifications with a JEOL 1230 electron microscope equipped with a 16 Mpixel CMOS TVIPS Temcam-F416 camera and operated at 100 kV. For image processing micrographs were captured at 40,000x nominal magnification (2.84 Å/pixel). For filament structural analysis, straight segments were manually selected and boxed at 200 × 200 and 128 × 128 pixels using EMAN^31^. For each sample, the extracted particles (CbTubZ GTP ~ 8000, CbTubZ GTP- γ-S ~ 4600, CbTubZ GDP ~ 7500, CbTubZ_E200A_ GTP ~ 20,000 and CbTubZ_T100A_ GTP ~ 12,500) were classified and averaged using the CL2D algorithm^32^. Helical reconstruction was performed with CbTubZ GTP data (128 × 128 pixels per particles) using the Iterative Helical Real Space Reconstruction Protocol (IHRSR^33^). An initial non-symmetrized three-dimensional reconstruction was performed using EMAN to obtain an initial estimation of the helical parameters, rise, and angle between monomers. A featureless cylinder of the appropriate diameter was used as starting model. Different values around the estimated ones were used as starting points in the IHRSR scripts to analyze parameter convergences. All runs converged on a similar final reconstruction. Rings were obtained from assembled TubZ_350_ in the presence of GTP and magnesium acetate after the addition of TubY_226_ (in a 1:1 ratio) to the solution. Images were selected manually and subsequently classified and averaged using XMIPP^34^.

### Atomic force microscopy

Samples of TubZ filaments were prepared as described above using a protein concentration of 10 μM in a Thermostat Plus (Eppendorf). For TubZ filaments coated with TubY, an additional 10 μM of TubY was added to the sample. Filaments were deposited onto freshly cleaved mica surface pre-treated with 20 μl MgCl_2_ (100 mM). After one minute of adsorption, the sample was gently washed with 3 ml Milli-Q water supplemented with 1 mM GTP and 6 mM magnesium acetate. GDP bound filaments were assembled as above, but with the addition of 20 μM wild type CbTubZ. Assembly time was set to 1 h and the mica was functionalized with 0.5% glutaraldehyde for the duration of the incubation time. GDP-bound filaments were adsorbed on the functionalized mica for 1 min and the sample was subsequently washed gently with 2 mL Milli-Q water supplemented with 2 mM GDP and 6 mM magnesium acetate. All AFM samples were dried by simple blotting; without using nitrogen gas. Samples were imaged in air at room temperature and low humidity using tapping mode with amplitude of 5 nm and a scan rate of 1 line·s^−1^ using an AFM from Nanotec (Nanotec Electrónica, Madrid, Spain). PointProbePlus were used (PPP-NCH, Nanosensors, Switwerland). Image processing consisted of a plane subtraction and flattening using the WSxM freeware^35^.

### Circular dichroism

Spectra were acquired at 25 °C using a 0.1 mm cell in a thermostated cell holder. Sample scanning conditions were as follows: 1 nm bandwidth; 1 nm measurement interval; 20 nm min^−1^ scan speed; 4 s time constant. Scans of each sample or buffer were repeated 4 times and the results averaged. CD data (millidegrees) were reduced to mean residue ellipticity values (degrees cm^2^ dmol^−1^) with the Jasco software and plotted.

### Crystallization and structure determination

TubZ_316_ crystals were grown at 22 °C in reservoir solution containing 0.1 M Na-citrate [pH 5.5], 0.1 M NaCl, 12% PEG4000 (Molecular Dimensions/MemStart-MemSys screen) in the presence of a peptide containing the last 8 residues of TubZ (NDFFSKYM) using the sitting-drop vapor-diffusion technique. Crystals were cryo-protected with 22% glycerol before being mounted in a nylon loop and flash-cooled in liquid nitrogen. X-ray diffraction data were collected at beamline PROXIMA 1 beamline (SOLEIL Synchrotron, France) using a wavelength 0.98011 Å and a DECTRIS PILATUS 6 M detector. Datasets were indexed, integrated, and scaled using XDS^36^ and the HKL reflection file was converted to a MTZ file using XDSCONV and the CCP4 suite supported program CAD^37^. Initial coordinates were determined by molecular replacement using PHASER^38^ and the coordinates for Apo-TubZ (Protein Data Bank (PDB) ID 3V3T). Ligand GDP coordinates and restrains were generated using ELBOW^39^ and fitted into the electron density maps using LigandFit^40^. No extra density was observed for the peptide. This was followed by several cycles of refinement using PHENIX^41^, which also was used for water picking, and iterative model building with COOT^42^. The final model was validated using MolProbidity^43^. Figures were generated using Pymol (Schrödinger).

### GTPase activity and polymer-bound nucleotide

GTP hydrolysis was monitored by taking 10 μL aliquots from protein polymerizing reactions at different times and measuring the release of inorganic phosphate in the malachite green assay at 650 nm^44^. For GTP-γ-S, hydrolysis and polymer-bound nucleotide samples were analyzed by HPLC^45^.

### *mant*-GTP binding affinity

Fluorescence anisotropy of free and protein-bound *mant*-GTP was measured with a spectrofluorimeter, with excitation and emission wavelengths of 357 nm (2 nm band pass) and 445 nm (5 nm band pass), respectively, using 2 × 10 mm cells at 25 °C. The binding of *mant*-GTP to TubZ causes a significant increase in its fluorescence that could lead to the calculation of erroneous apparent Kb. To avoid this error, the ratios between the fluorescence intensities of TubZ-bound and free *mant*-GTP were determined from the different constructs (R_TubZ_ = 2.2; R_TubZ350_ = 2.7; R _TubZ331_ = 2.7; R_TubZ316_ = 2.4) and used to correct all anisotropy-based binding calculations. To measure the Kb, *mant*-GTP (500 nM) was titrated with TubZ proteins at different concentrations (0.075–10 μM) in PKE buffer. The converged data were used to calculate the binding constant^46^.

## Additional Information

**Accession codes** (Protein Data Bank 4XCQ).

**How to cite this article:** Fuentes-Pérez, M. E. *et al*. TubZ filament assembly dynamics requires the flexible C-terminal tail. *Sci. Rep.* **7**, 43342; doi: 10.1038/srep43342 (2017).

**Publisher's note:** Springer Nature remains neutral with regard to jurisdictional claims in published maps and institutional affiliations.

## Supplementary Material

Supplementary Information
