# Supplementary material for: TubZ filament assembly dynamics requires the flexible C-terminal tail
Source: Sci Rep. 2017 Feb 23;7:43342. doi: 10.1038/srep43342 (PMC5322520; doi:10.1038/srep43342)
Supplement: Supplementary Information [file srep43342-s1.pdf]

Supplemental Information

TubZ filament assembly dynamics requires the flexible C-terminal tail.

María E. Fuentes-Pérez[2,3], Rafael Núñez-Ramírez[1], Alejandro Martín-González[2], David Juan-Rodríguez[1], Oscar Llorca[1], Fernando Moreno-Herrero[2], María A. Oliva[1]*.

[1] CSIC – Centro de Investigaciones Biológicas, Department of Chemical and Physical Biology. Madrid, 28040, Spain

[2] CSIC – Centro Nacional de Biotecnología, Department of Macromolecular Structures, Cantoblanco - Madrid, 28049, Spain

[3] Imperial College, London, W120NN, UK

*Correspondence: marian@cib.csic.es

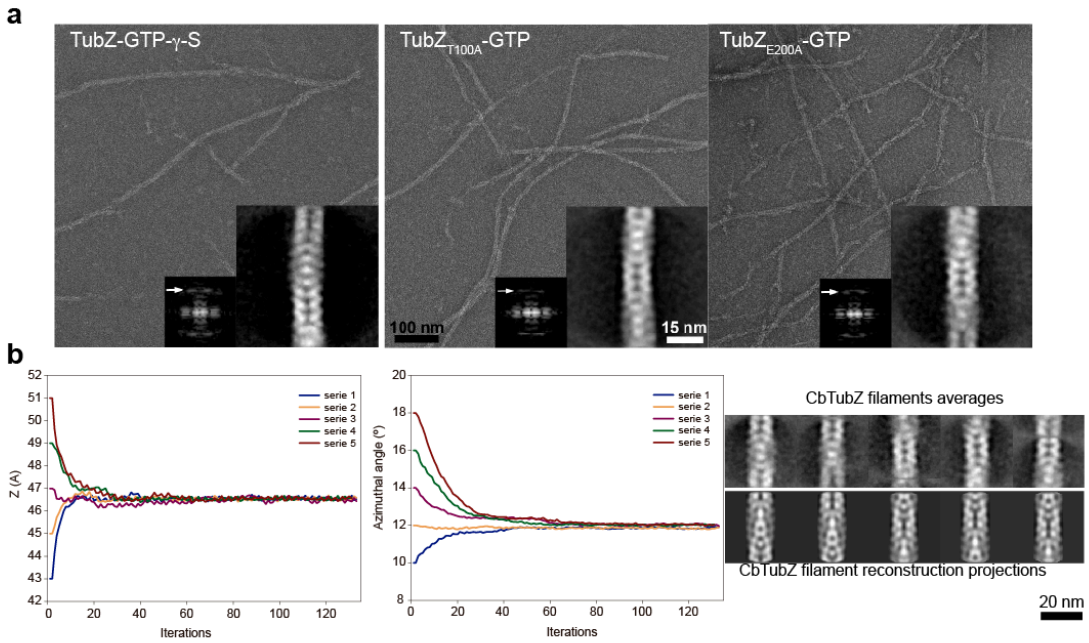

Figure S1: a. Negative stain EM showing wild type CbTubZ filaments grown in the presence of GTP-γ-S, and $CbTubZ_{T100A}$ and $CbTubZ_{E200A}$ assembled in the presence of GTP. Insets correspond to the averaged filaments and its Fourier transform, where arrow indicates a 4.8 nm longitudinal spacing between molecules **b.** Refinement of the filament helical Z value (left) and the azimuthal angle (middle) showing the convergence to ~46 Å and ~12º when starting from very different points. Further, the projections of the reconstructed filament are very similar to the experimental filament averages (right)

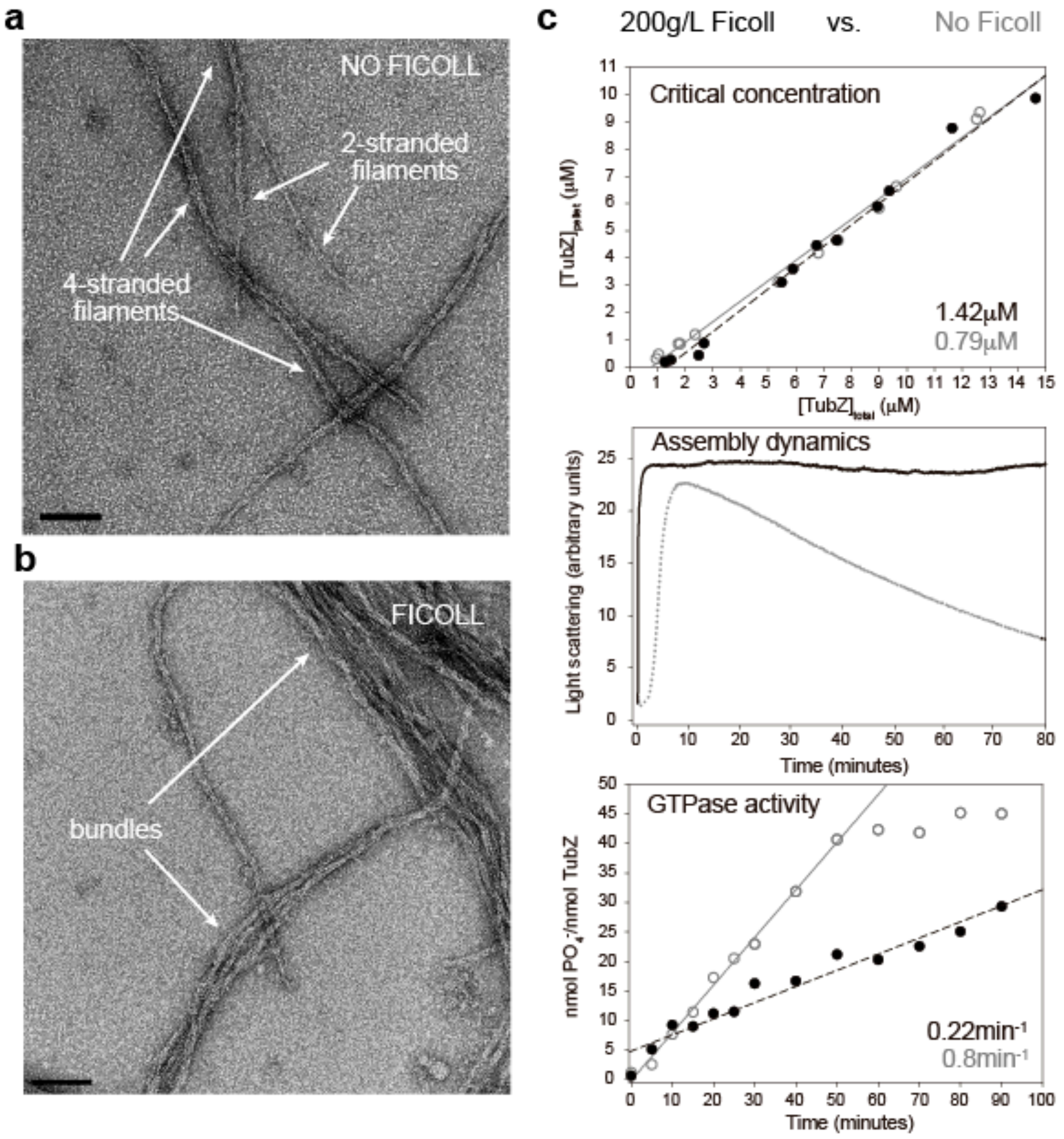

Figure S2: a-b. EM images of wild type CbTubZ filaments assembled with GTP/Mg^{2+} in diluted buffer and in buffer with Ficoll (200 g/L), showing 2- and 4-stranded filaments and the bundling trend (bars 90 nm). **c.** Critical concentration measurements, light scattering profiles and GTPase activity determination in in buffer with Ficoll (black) vs. diluted buffer (grey).

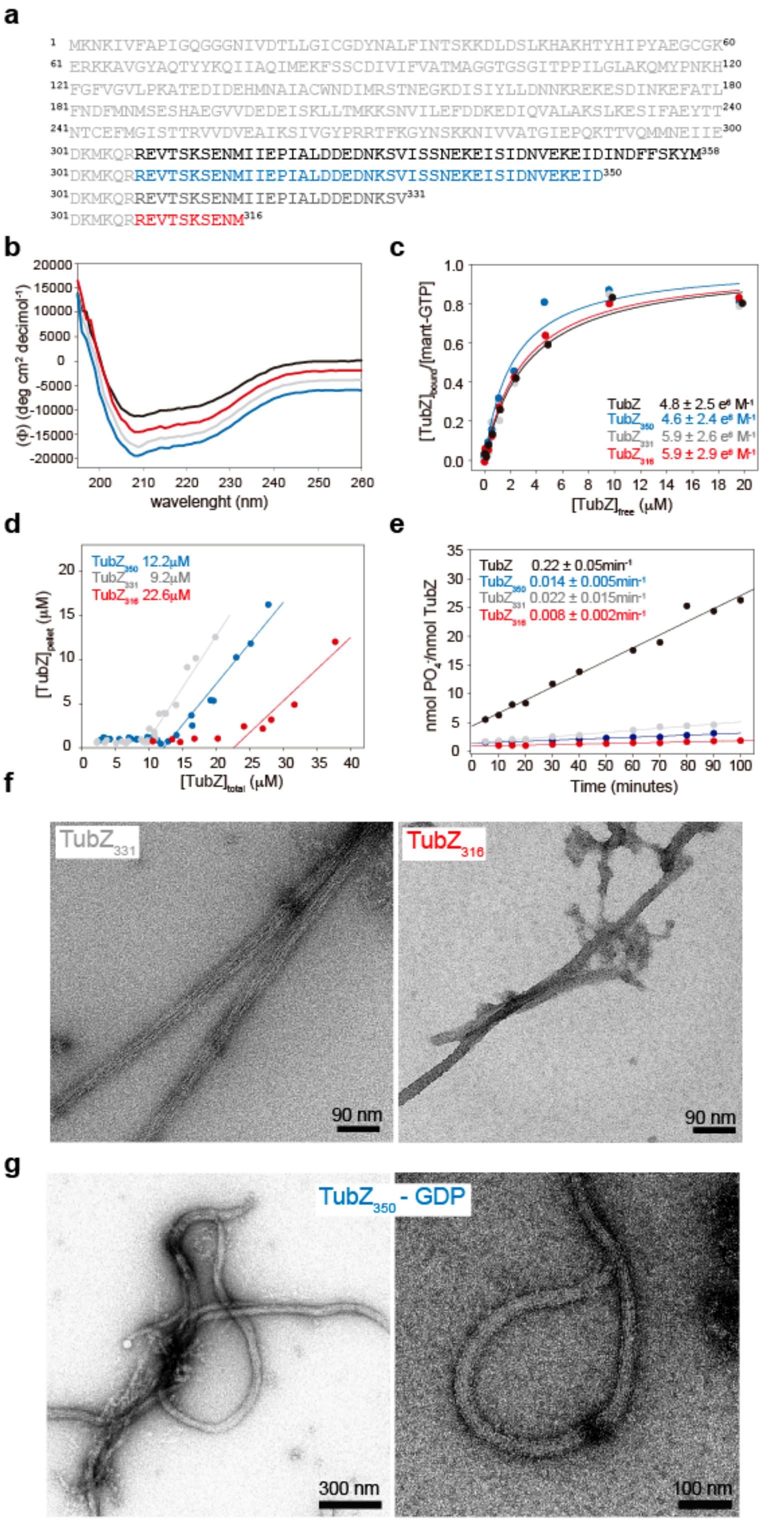

Figure S3: a. Sequences of wild type and C-tail truncated constructs. Light grey denotes the known structure and colors refer to the C-tail sequence in each different construct: wild type (black), $TubZ_{350}$ (blue), $TubZ_{331}$ (grey) and $TubZ_{316}$ (red). The coloring is conserved along all figures in this study **b.** Circular dichroism spectra showing similar secondary structure composition. The lines have been shifted 2000 units in the Y-axis in order to distinguish them **c.** GTP binding affinity (K_B) calculated using GTP fluorescent analog mant-GTP **d**. Critical concentration measurements **e.** GTPase activity analysis using the malachite green assay **f-g.** Negative stain EM of $CbTubZ_{331}$ and $CbTubZ_{316}$ stiff filament assembled in the presence of GTP/Mg^{2+} (f) and $CbTubZ_{350}$ flexible filaments polymerized with GDP/Mg^{2+} (g)

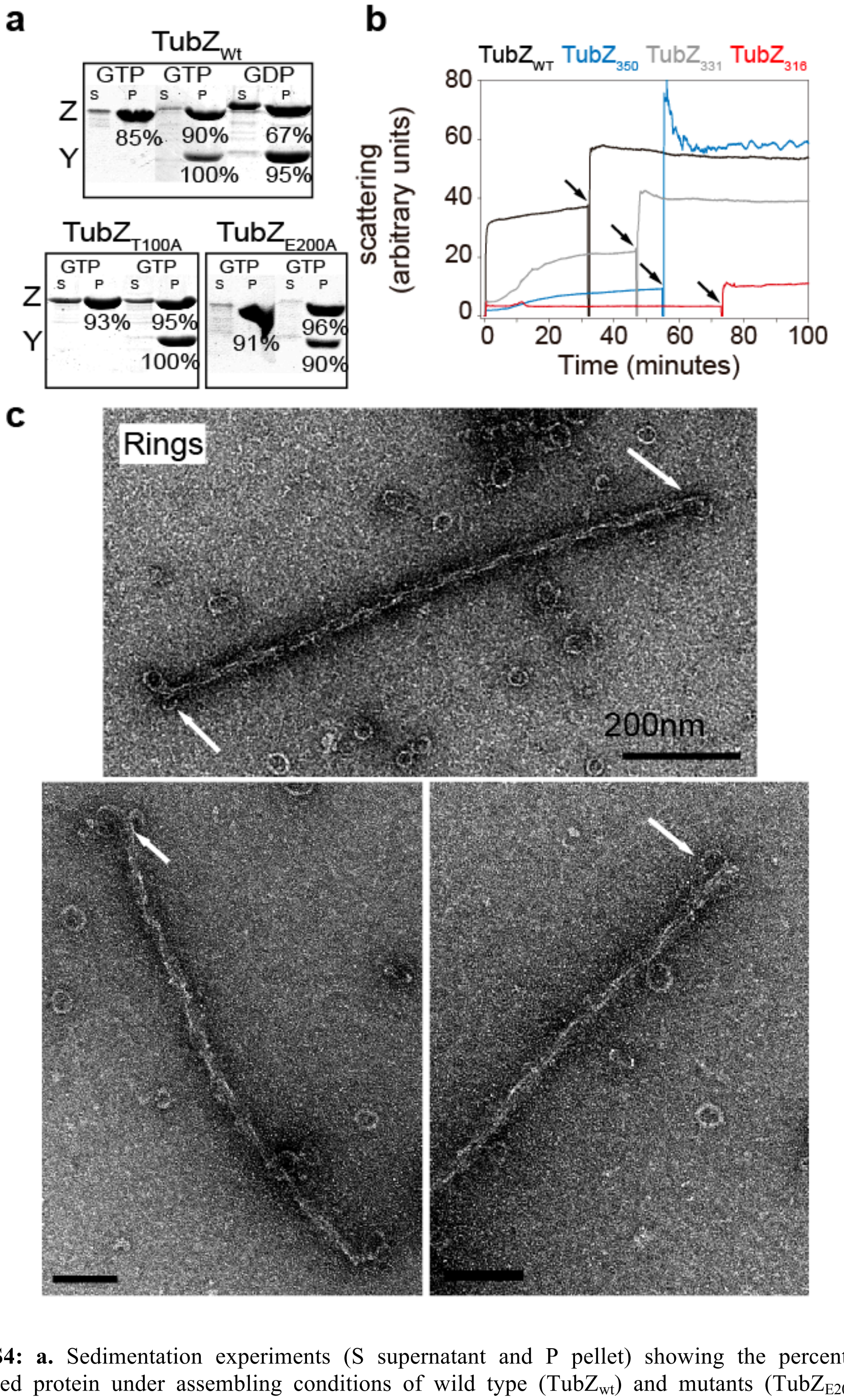

Figure S4: a. Sedimentation experiments (S supernatant and P pellet) showing the percentage of precipitated protein under assembling conditions of wild type ($TubZ_{wt}$) and mutants ($TubZ_{E200A}$ and $TubZ_{T100A}$) in the presence or absence of TubY. The co-sedimentation denotes interaction since TubY alone did not precipitate under those conditions (Oliva et al., 2012) **b.** Light scattering of proteins assembled at a concentration 8-fold above the critical concentration, showing the increase of the scattered signal when TubY is added to pre-assembled wild type (black), $TubZ_{350}$ (blue), $TubZ_{331}$ (grey) and $TubZ_{316}$ (red) filaments. Arrows points to the moment TubY was added **c.** EM images showing the detail of rings formation upon disassembly from filament tips when $TubZ_{350}$ filaments are in the presence of TubY.

Table S1: Data collection and refinement

		Native $TubZ_{316}$-GDP (4XCQ)
**Data collection**		
Space group		C2
Unit cell parameters	*a, b, c* (Å)	104.970, 86.342, 44.734
	α, β, γ (°)	90.00, 92.49, 90.00
Resolution range (Å)		44.69 - 2.39
No. of reflections*		104624 (14464)
No. of unique reflections*		15852 (2479)
Completeness (%)*		99.4 (96.8)
Redundancy*		6.6 (5.8)
C (1/2)*		99.1 (48.9)
I/σ (I)*		7.7 (0.98)
**Refinement**		
No. reflections		15655
R_{work}/ R_{free}[a]		0.19/0.24
No. atoms		
Protein		2381
Water		43
Ligand (GDP)		28
B-factor		
Protein		51.20
Water		46.33
r.m.s deviation		
Bond lengths (Å)		0.009
Bond angles (°)		1.181

* Highest resolution shell is shown in parenthesis

SUPPLEMENTAL REFERENCES

Oliva, M.A., Martin-Galiano, A.J., Sakaguchi, Y., and Andreu, J.M. (2012). Tubulin homolog TubZ in a phage-encoded partition system. Proceedings of the National Academy of Sciences of the United States of America *109*, 7711-7716.
